# Reduced PKC *α* Activity Induces Senescent Phenotype in Erythrocytes

**DOI:** 10.1155/2012/168050

**Published:** 2012-09-04

**Authors:** Rukmini B. Govekar, Poonam D. Kawle, Suresh H. Advani, Surekha M. Zingde

**Affiliations:** ^1^Advanced Centre for Treatment, Research and Education in Cancer, Tata Memorial Centre, Kharghar, Navi Mumbai 410610, India; ^2^Department of Medical Oncology, Jaslok Hospital and Research Centre, Mumbai 400026, India

## Abstract

The molecular mechanism mediating expression of senescent cell antigen-aggregated or cleaved band 3 and externalized phosphatidylserine (PS) on the surface of aged erythrocytes and their premature expression in certain anemias is not completely elucidated. The erythrocytes with these surface modifications undergo macrophage-mediated phagocytosis. In this study, the role of protein kinase C (PKC) isoforms in the expression of these surface modifications was investigated. Inhibition of PKC *α* by 30 **μ**M rottlerin (R30) and 2.3 nM Gö 6976 caused expression of both the senescent cell marker-externalized PS measured by FACS analysis and aggregated band 3 detected by western blotting. In contrast to this observation, but in keeping with literature, PKC activation by phorbol-12-myristate-13-acetate (PMA) also led to the expression of senescence markers. We explain this antithesis by demonstrating that PMA-treated cells show reduction in the activity of PKC *α*, thereby simulating inhibition. The reduction in PKC *α* activity may be attributed to the known downregulation of PMA-activated PKC *α*, caused by its membrane translocation and proteolysis. We demonstrate membrane translocation of PKC *α* in PMA-treated cells to substantiate this inference. Thus loss of PKC *α* activity either by inhibition or downregulation can cause surface modifications which can trigger erythrophagocytosis.

## 1. Introduction

Human erythrocytes have a definite lifespan of 120 ± 4 days in circulation and thereafter are marked for phagocytosis by cell surface modifications, such as aggregation or cleavage of protein band 3 and exposure of PS [1–3]. Time-compressed expression of these markers leads to premature eryptosis in anemias [4, 5]. Molecular events which mediate expression of these surface markers of senescence have been partly delineated in erythrocytes mainly under oxidative conditions [6, 7]. They appear to recapitulate the cytoplasmic events in apoptosis of nucleated cells such as translocation of Fas into rafts, formation of a Fas-associated complex, and activation of caspases 8 and 3 [8]. Activation of caspase 3 in turn is associated with cleavage of band 3 [9], which generates senescent cell antigen in erythrocytes [10], as well as causes impairment of aminophospholipid flippase activity and PS externalization [11]. This similarity of molecular events in eryptosis and apoptosis prompted us to explore the role of PKC isoforms, which have distinct tissue-specific roles in both cell survival and apoptosis of nucleated cells [12], in eryptosis.

 PKC is a family of serine/threonine kinases comprising of eleven isoforms which differ in their cofactor requirement for activation and are accordingly categorized into classical (Ca^2+^/diacylglycerol (DAG) dependent: *α*, *β*I, *β*II, *γ*), novel (DAG dependent: *δ*, *ε*, *η*, *θ*), and atypical (Ca^2+^/DAG independent: *ζ*, *ι*, related kinase *μ*) isoforms [13]. We have earlier shown that normal erythrocytes express PKC *α*, *ζ*, *ι*, *μ* [14]. This investigation demonstrates in vitro that reduction in the activity of PKC *α* causes expression of the senescent cell antigens in erythrocytes.

## 2. Materials and Methods

### 2.1. Chemicals

PMA (P 8139), 4*α*-phorbol 12,13-didecanoate (4*α*PDD) (P 8014), phenyl methyl sulfonyl fluoride (PMSF) (P 7626), anti-band 3 N-terminus monoclonal antibody (B 9277), and anti-*β*-actin antibody (A5316) were purchased from Sigma. Rottlerin (557370) and Gö 6976 (365250) were procured from Calbiochem. PKC activity kit (RPN 77), enhanced chemiluminescence reagent (ECL plus) (RPN 2132), and the horseradish-peroxidase (HRP) conjugated-anti-mouse IgG (NA 931) were from GE Healthcare. Polyvinylidene difluoride (PVDF) membrane (IPVH 00010) was from Millipore. Annexin V-FITC apoptosis detection kit I (556547) was from BD Pharmingen. Anti-PKC *α* antibody was from PKC sampler kit (S 85080) of the Transduction Laboratories. Colloidal gold total protein stain (170-6527) was from Bio-Rad laboratories. *γ* ^32^P ATP was obtained from Board of Radiation and Isotope Technology, Department of Atomic Energy, India.

### 2.2. Biological Material

This study was undertaken after obtaining ethics clearance from the hospital ethics committee, and informed consent form was administered prior to sample collection. Peripheral blood (5 mL) was collected by venipuncture in ethylene diamine tetra acetic acid (EDTA)-containing bulbs for separation of erythrocytes. Healthy voluntary donors (*N*) who reported no health problems were recruited for the study (*n* = 20). The age of volunteers ranged from 22 to 56 years. An equal number of male and female volunteers in the age ranges 20–30, 30–40, 40–50, and 50–60 years were included.

### 2.3. Preparation of Erythrocyte Suspension

Erythrocytes were allowed to settle from the blood sample collected in EDTA bulbs. After removing the supernatant plasma, erythrocytes were washed three times in wash buffer (10 mM Tris pH 7.6, 150 mM NaCl) separating erythrocytes each time by centrifugation at 1500 rpm for 15 min at 4°C.

### 2.4. Treatment of Erythrocytes with Activators and Inhibitors of PKC

Erythrocytes (10^8^ cells/mL) were incubated at 37°C for 20 min with either dimethylsulfoxide (DMSO)—solvent for all the modifiers (final concentration 1.6% as it was the highest concentration in the inhibitor/activator treated groups), 1 *μ*M PMA-activator of classical and novel isoforms of PKC, 1 *μ*M 4*α*PDD—biologically inactive structural analogue of PMA, 30 *μ*M rottlerin (R30)—inhibitor of PKC (inhibits classical isoforms *α*,  *β*, and  *γ* and novel isoforms *δ* and *θ* at the concentration used but not atypical isoforms PKC *ζ*,  *ι* or *μ*). This treatment group comprising of ten samples was used for the initial assessment of the role of PKC in the aggregation of band 3 and PS externalization as well as to assess membrane translocation of PKC *α*. To confirm the role of PKC *α* in expression of markers of senescence, aggregation of band 3 and externalized PS were detected in erythrocytes from ten additional samples incubated with DMSO or 2.3 nM Gö 6976—a specific inhibitor of PKC *α*. After treatment with modifiers the cells were aliquoted.

### 2.5. Analysis of PS Externalization

Aliquots of cells (1 × 10^7^) treated with modifiers and untreated controls were labelled with annexinV-FITC to detect PS exposure on erythrocytes. Labelling was performed using the annexinV-FITC apoptosis detection kit according to the manufacturer's instructions. Data acquisition was performed on a Becton Dickinson FACS Calibur flow cytometer, and analysis was done with Cell Quest software. Per sample 10,000 events were acquired. The percentage of annexin V-positive erythrocytes from the gated population was determined for each treatment group and compared with a negative (unlabeled) control, which was run for each sample.

### 2.6. Preparation of Erythrocyte Lysates for Western Blotting

An aliquot of the erythrocytes treated with modifiers or DMSO was lysed in equal volume of hypotonic solution (10 mM Tris pH 7.6, 1 mM EDTA, 20 *μ*g/mL PMSF) as described by Dodge et al. [15]. After centrifugation of the lysate at 15,000 rpm for 15 min at 4°C in SS-34 rotor of Sorvall RC-5C centrifuge, the supernatant was recovered as cytosolic fraction and the pellet which contained the membrane skeleton (referred to as “membrane”) was washed thrice with wash buffer (10 mM Tris pH 7.6, 150 mM NaCl, 20 *μ*g/mL PMSF). Membrane and cytosol fractions were aliquoted and preserved at −70°C until use. Protein was estimated using the modified Lowry's method [16]. 

### 2.7. Detection of Band 3 and Translocation of PKC *α*

Erythrocyte membrane protein (60 *μ*g) and cytosolic protein (120 *μ*g) obtained from cells treated with DMSO and modifiers were resolved on 10% SDS-polyacrylamide gels and then transferred electrophoretically to PVDF membrane [17]. The blots with membrane proteins were probed with anti-band 3 antibody. The protein-antibody complexes were further reacted with anti-mouse IgG-HRP, and the binding of HRP-labelled antibody was detected by autography using the ECL plus western blotting detection system. A duplicate blot was used for probing with antibody to *β*-actin, and the recorded signals were assessed to ascertain equal loading. For the samples with quantities inadequate for duplicate blot, the blot stained with band 3 antibody was stained with colloidal gold to assess equal loading of protein. Western blots of both membrane and cytosolic proteins from cells treated with DMSO, PMA, and 4*α*PDD were probed with PKC *α* antibody to assess translocation of PKC *α* from cytosol to membrane. Detection of protein-antibody complex was done similar to that for band 3.

### 2.8. Preparation of Whole Cell Lysates for the PKC Activity Assay

Erythrocytes (1–3 × 10^9^) were lysed in 1 mL cold cell lysis buffer (50 mM HEPES pH 7.6, 150 mM NaCl, 10% glycerol, 1% Triton X-100, 1.5 mM MgCl_2_, 5 mM EGTA, 1 mM leupeptin, 2 mM PMSF, 10 *μ*g/mL aprotinin, 10 *μ*g/mL pepstatin, 1 mM sodium orthovanadate, and 1.5 mM sodium fluoride). The mixture was kept on ice for 15 min. and spun at 1,00,000 g for 30 min. in a Beckman TL-100 ultracentrifuge. After adding an equal volume of glycerol to the supernatant, it was stored at −70°C till use.

### 2.9. Estimation of Activity of PKC *α*

PKC activity was determined using the PKC enzyme assay system as per manufacturer's instructions. Incorporation of *γ*-^32^P-labelled ATP (45,000 ± 20,000 cpm in magnesium buffer per reaction) in PKC-specific substrate was measured in total lysates (25 *μ*L) (of DMSO- and PMA-treated erythrocytes) incubated with Ca^2+^ and lipid (as a mixture of *α* phosphatidyl-L-serine and phorbol-12-myristate-13-acetate in the kit). Incorporation of labelled ATP in the peptide substrate was measured by liquid scintillation counting. Activity was expressed as pmol phosphate transferred/min/mg protein.

### 2.10. Statistical Analysis

Comparison of PS externalization observed in erythrocytes treated with different modifiers was expressed as mean ± standard error (SE) for the specified number of samples (*n*) and analyzed by either paired *t*-test or Wilcoxon signed ranks test as indicated, using SPSS software version 15.

## 3. Results and Discussion

**Figure 1:**
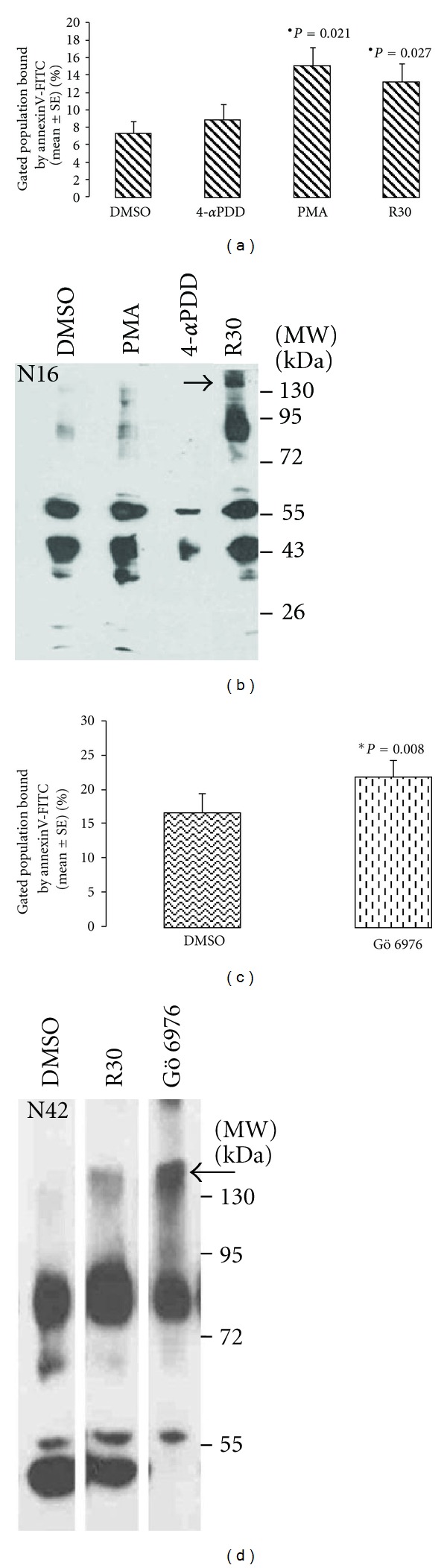
Externalization of PS and aggregation of band 3 induced by PKC *α* activator (PMA) and inhibitors (30 *μ*M rottlerin-R30 and Gö 6976). Flow cytometry of annexinV-bound cells shows significant (*) increased percentage of cells with externalized PS upon treatment with (a) PMA, R30 (Wicoxon signed rank test; *n* = 10) as well as (c) with Gö 6976(paired *t*-test, *n* = 10). A signal for aggregated band 3 above 130 kDa (indicated by arrow) is seen in western blot of erythrocyte membrane proteins immunostained with band 3 antibody only in cells treated with (b) R30 (represented in sample *N*16) and (d) Gö 6976 (represented by *N*42).

We have earlier demonstrated that PKC *α* is the only DAG-dependent and thus PMA-activated PKC isoform expressed in erythrocytes, while PKC *ζ*, *ι*, and *μ* are atypical isoforms which are non-responsive to PMA [14]. Thus the significant increase (*P* = 0.021; Wilcoxon Signed Ranks Test) in cells expressing externalized PS upon the activation of PKC with PMA (Figure 1(a)) can be attributed to PKC *α*. This is in keeping with the literature reports on PKC-induced PS externalization [18, 19] as well as its attribution to PKC *α* [20]. In cells treated with 4*α*PDD, a biologically inactive structural analogue of PMA, the percentage of cells expressing PS remained unchanged. The effect of PMA was less obvious on aggregation of band 3 (Figure 1(b)).

The conclusion of causative role of PKC *α* activation in externalization of PS was differed by our observation in the inhibition experiments. Preferential inhibition of PKC *α* was achieved by using 30 *μ*M rottlerin which inhibits PKC *α* while the atypical isoforms *ζ*, *ι*, and *μ* are inhibited by 80–100 *μ*M [21]. In erythrocyte samples (*n* = 10) treated with 30 *μ*M rottlerin, significant PS externalization (*P* = 0.027; Wilcoxon Signed Ranks Test) (Figure 1(a)) as well as aggregation of band 3 (in 9/10 samples) was observed (represented in Figure 1(b)). The role of PKC *α* inhibition was confirmed by demonstrating expression of both the markers of senescence (Figures 1(c) and 1(d)) in erythrocytes treated with 2.3 nM Gö 6976, which specifically inhibits PKC *α* [22]. These observations are significant in the light of the reported [23] loss of PKC activity in senescent erythrocytes. 

**Figure 2:**
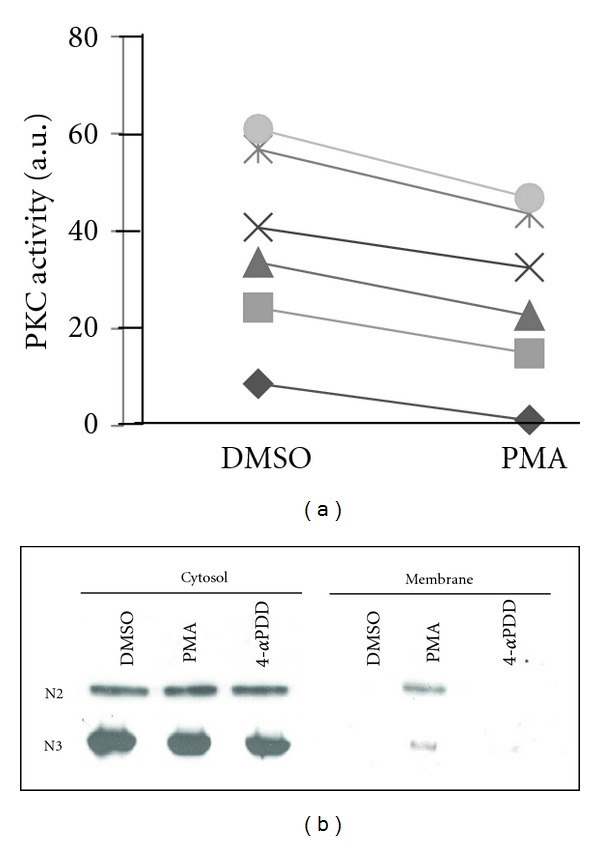
Modulation of PKC *α* localization and activity upon PMA treatment. (a) Activity of PKC in the presence of Ca^2+^ and lipid shows 10–30% reduction in PMA-treated group. Units of activity (a.u.) are arbitrary values assigned by the graphic tool. (b) Western blots of erythrocyte cytosolic and membrane proteins stained with anti-PKC *α* antibody (represented by *N*2 and *N*3) show a signal around 77 kDa in the cytosol of all treatment groups but in the membrane fractions of only the PMA-treated erythrocytes.

Generation of similar responses with agents which activate (PMA) or inhibit (rottlerin) PKC in an isoform-non-specific manner is also reported by Liu et al. [24]. We explain this antithesis by the demonstration of 10–30% reduction in PKC *α* activity (Figure 2(a)) in the presence of Ca^2+^ and DAG (which activate only PKC *α* in erythrocyte) in PMA-treated cells as compared to control. We show translocation of activated PKC *α* (Figure 2(b)) in PMA-treated cells which is known to cause their downregulation by calpain-mediated proteolysis [25]. Translocation is not observed in samples treated with DMSO or 4*α*PDD. In erythrocytes which do not synthesize protein, downregulation would lead to permanent loss of activatable protein, thereby simulating conditions of inhibition. We therefore redefine the mechanism of PMA-mediated expression of externalized PS in erythrocytes as caused by loss of PKC *α* activity due to downregulation rather than activation of the molecule. 

**Figure 3:**
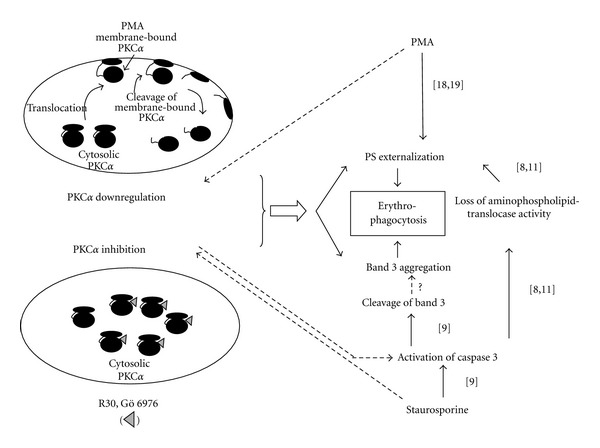
PKC *α* in eryptosis. Literature reports the role of activation of PKC/PKC *α* in the expression of externalized PS. We demonstrate that loss of PKC *α* activity due to inhibition or activation-linked down fregulation causes expression of not only externalized PS but also aggregated band 3. These observations along with other reports (given in brackets along the arrows) are linked to further understand the molecular mechanism of eryptosis. The molecular pathway of eryptosis emerging from the reports and the present study suggests exploration of the role of PKC *α* inhibition in activation of caspase 3 (indicated by dotted lines) which causes expression of both the markers of senescence.

Thus we report for the first time that loss of PKC *α* activity due to inhibition or downregulation causes expression of both markers of erythrocyte senescence which can mediate erythrophagocytosis.  Figure 3 highlights the insights provided by this finding into the aspects of PMA-mediated molecular events in erythrocyte aging reported in literature. 

## 4. Conclusion

Thus, PKC isoforms, which have distinct tissue-specific roles in both cell survival and apoptosis of nucleated cells, can also mediate eryptosis. While literature reports the role of activation of PKC/PKC *α* in the expression of externalized PS, we demonstrate that loss of PKC *α* activity due to inhibition or activation-linked downregulation can cause expression of not only externalized PS but also aggregated band 3. The study has thus unravelled a molecular event causative of the expression of two cell surface modifications, which can trigger erythrophagocytosis.
